# An analysis of 37 patients with uterine leiomyosarcoma at a high-volume cancer center

**DOI:** 10.4274/tjod.33602

**Published:** 2015-09-15

**Authors:** Ulaş Solmaz, Levent Dereli, Gülşah Selvi Demirtaş, Atalay Ekin, Emre Mat, Cenk Gezer, Pınar Solmaz Hasdemir, Sevil Sayhan, Muzaffer Sancı, Niyazi Aşkar

**Affiliations:** 1 Tepecik Education and Research Hospital, Clinic of Gynecologic Oncology, İzmir, Turkey; 2 Tavas State Hospital, Clinic of Obstetrics and Gynecology, Denizli, Turkey; 3 Tepecik Education and Research Hospital, Clinic of Pathology, İzmir, Turkey; 4 Ege University Faculty of Medicine, Department of Obstetrics and Gynecology, İzmir, Turkey

**Keywords:** Uterine leiomyosarcoma, lymph node dissection, stage, Survival

## Abstract

**Objective:**

To evaluate the clinicopathologic characteristics, treatment methods, survival, and prognosis of uterine leiomyosarcoma (ULMS).

**Materials and Methods:**

All patients with ULMS who were treated between January 1998 and October 2012 were retrospectively reviewed. A total of 37 women who met the inclusion criteria were included in the present study. Univariate and multivariate analyses were used to identify the risk factors for overall survival (OS) and progression-free survival (PFS).

**Results:**

The majority of patients had stage 1 disease (IA, n=9 (24.3%); IB, n=23 (62.1%)). All patients underwent total abdominal hysterectomy and bilateral salpingo-oophorectomy. Additionally, only pelvic, and pelvic plus para-aortic lymphadenectomy was performed in 5 (13.5%) and 8 (21.6%) women, respectively. Adjuvant treatment was administered to 27 (72.9%) patients. Patients who did not receive adjuvant therapy had stage 1 disease. Recurrences occurred in 5 (13.5%) patients. The median follow-up period was 71 months (range 1-158 months). The 5-year PFS and OS rates were 68% and 74%, for all patients. The 5-year OS rates for women with stage 1 and ≥ stage 2 disease were 82% and 27%, respectively. Multivariate analysis confirmed stage 1 disease as the only independent predictor of both PFS (Odds ratio (OR) 10.955, 95% confidence interval (CI) 1.686-71.181, (p=0.012)) and OS (OR 57.429, 95% CI 3.287-1003.269, (p=0.006)).

**Conclusions:**

Extensive surgery is not associated with prognosis and stage 1 disease is the only independent good prognostic factor for survival in patients with ULMS.

## INTRODUCTION

Uterine leiomyosarcomas (ULMSs) are rare malignancies, which constitute approximately 1% to 3% of all uterine cancer types^(1,2,3)^. These tumors are associated with an aggressive clinical course and poor prognosis. Even though these tumors are usually confined to the uterus at the time of diagnosis, high recurrence rates ranging from 45% to 73% have been reported^(2,3,4)^. The reported 5-year overall survival (OS) rates range from 30% to 42%^(2,3,4,5)^.

Total abdominal hysterectomy (TAH) is the standard approach as an initial therapy for ULMS but the need for additional surgical procedures such as bilateral salpingo-oophorectomy (BSO), pelvic (P) and/or para-aortic (PA) lymphadenectomy and adjuvant therapy is a widely debated issue^(5,6)^. Neither surgical treatment nor postoperative therapy protocols have been completely standardized. In the present study, we evaluated the clinico-pathologic characteristics, prognostic factors, and treatment strategies in 37 patients with ULMS.

## MATERIALS AND METHODS

### Patients

All patients with ULMS who underwent surgery between January 1998, and October 2012, were retrospectively reviewed. This study was performed in accordance with the ethical standards of the Declaration of Helsinki and was approved by the local ethics committee of our institution. Patients who did not undergo surgery and patients with missing data were excluded. Patients with any other primary cancer were not included in the study.

### Data collection

Demographic data, such as age at diagnosis, parity, surgical and adjuvant treatment details, and follow-up information, were obtained from medical records. Histopathologic findings, including primary tumor diameter (PTD), depth of myometrial invasion (MI), lymphovascular space invasion (LVSI), P and/or PA lymph node involvement, mitotic counts, cellular atypia, tumor grade, tumor cell necrosis, and the size and location of extra-uterine metastatic tumors were retrieved from surgical pathology reports. All of the pathology slides were reviewed by an experienced gynecologic pathologist using the criteria proposed by Bell et al.,^(7)^ which include the degree of atypia, the presence of necrosis, and mitotic counts.

### Surgical technique

All patients underwent laparotomy. Fluid from peritoneal washing was obtained during surgery for cytologic analysis. TAH with BSO was performed in all patients (some of the patients who underwent myomectomy at initial operation were subsequently re-operated a few weeks later after histologic confirmation of leiomyosarcoma). During the study period, the decision to perform a systematic P and PA lymphadenectomy was made at the surgeon’s discretion; no lymph nodes were sampled in some patients, only the P or PA nodes were sampled in some patients, complete staging with bilateral P lymph node dissection (LND) was applied in some patients, and some patients underwent complete staging with bilateral P and PA LND. Individual practitioners were responsible for these variations over the study period. Staging criteria were determined postoperatively based on the 2009 International Federation of Gynecology and Obstetrics (FIGO) staging system.

### Adjuvant treatment

Adjuvant therapy, including chemotherapy (CT) alone, radiotherapy (RT, including internal radiotherapy (IRT) and external radiotherapy (ERT)) alone, or a combination of both, was administered to patients based on stage, age, nodal metastasis status, performance status, and the presence/absence of medical comorbidities. The CT regimens were as follows: the ifosfamide, mesna and adriamycin (IMA) regimen, ifosfamide-based regimen, and adriamycin-based regimen. All three regimen were administered intravenously every 21 days to a maximum of 6 cycles. Mesna was given as an intravenous bolus at a dose of 400 mg/m^2^ before ifosfamide therapy. Ifosfamide and adriamycin were given at a dose of 2500 mg/m^2^ and 60 mg/m^2^, respectively. ERT was administered at a median dose of 50.4 Gy (range, 45-54 Gy) in 1.8-2.0 Gy per fraction, 5 days a week. IRT (2x6.5 Gy and 3x6 Gy when combined with ERT; 3x7 Gy when applied as the sole RT modality) was delivered via a vaginal applicator fitted with a source of high dose-rate iridium-192.

### Clinical follow-up

The patients returned for follow-up evaluations every 3 months for the first 2 years, every 6 months for the next 3 years, and annually there after. Follow-up evaluations consisted of physical and vaginal examinations, vaginal cytology, ultrasound scanning and assessment of serum CA 125 values. Computed tomography or magnetic resonance imaging was performed annually. Progression-free survival (PFS) was defined as the time from the date of primary surgery to the detection of recurrence or the latest observation. OS was defined as the time interval from the date of surgery to death or last contact.

### Statistical analysis

Statistical analyses were performed using IBM SPSS Statistics 22.0 (SPSS Inc., Chicago, IL). The variables were assessed using visual (histograms, probability plots) and analytical methods to determine whether they were normally distributed. Continuous data (presented as the mean ± SD and median (min-max)) were analyzed using the Mann-Whitney U test for non-normal data. The Chi-Square test (Pearson’s Chi-Square and Pearson’s Exact Chi-Square tests) was used to compare the proportions between groups. Univariate and multivariate logistic regression models were used to identify risk factors. The Kaplan-Meier method was used to generate the survival curve, and comparisons were performed with the log rank test. A p-value <0.05 was defined as statistically significant.

## RESULTS

A total of 37 patients with UMLS who fulfilled the inclusion criteria were included. The median age at diagnosis was 52 years (range, 32-71 years), and 25 (67.6%) patients were postmenopausal. Abnormal vaginal bleeding (67.6%) was the commonest presentation. Patients had no history of pelvic irradiation before diagnosis. Thirty-two patients (86.5%) presented with FIGO stage 1 disease (IA, n=9 (24.3%); IB, n=23 (62.1%)), 2 (5.4%) with stage 2 disease, and 3 (8.1%) with stage 4 disease. The demographic and clinico-pathologic characteristics are summarised in Table 1.

A total of 28 (75.7%) patients had a history of diagnostic dilatation and curettage (D&C) preoperatively, and only 3 had malignant histopathology. Of the remaining 9 (24.3%) patients who did not undergo D&C; 3 (8.1%) had pathologic confirmation after myomectomy, and 31 (83.7%) patients were diagnosed as having leiomyosarcoma after hysterectomy was performed for suspected benign disease. Among the 37 patients, 24 (64.9%) underwent TAH+BSO, 5 (13.5%) underwent TAH+BSO and P lymphadenectomy, and 8 (21.6%) underwent TAH+BSO and P plus PA lymphadenectomy. Adjuvant treatment was administered to 27 patients (72.9%); 2 patients (5.4%) received CT alone, 13 patients (35.1%) received only RT, and 12 (32.4%) received both RT and CT. The 10 (27.1%) patients who did not receive adjuvant therapy had stage 1 disease.

PTD were <5 cm in 8 (21.6%) patients, >10 cm in 12 (32.4%) patients, and between 5-10 cm in 17 (45.9%) patients. Tumor necrosis was present in 19 (51.4%) patients. Two (13.5%) patients had lung metastasis alone, and 1 patient had both inguinal lymph node and lung metastasis.

In the present study, the univariate analysis found that stage (stage 1 vs. stage ≥2), and absence of tumor necrosis were the significant prognostic factors for PFS (p=0.04, and p=0.03, respectively). Adjuvant therapy (yes vs. no) was the only significant prognostic factor for both PFS and OS (p=0.001, and p=0.045, respectively). Multivariate analysis confirmed that disease confined to the uterus (stage 1) was the only independent predictor of both PFS (Odds ratio (OR) 10.955, 95% confidence interval (CI) 1.686-71.181, (p=0.012)) and OS (OR 57.429, 95% CI 3.287-1003.269, (p=0.006)).

**Figure 1:**
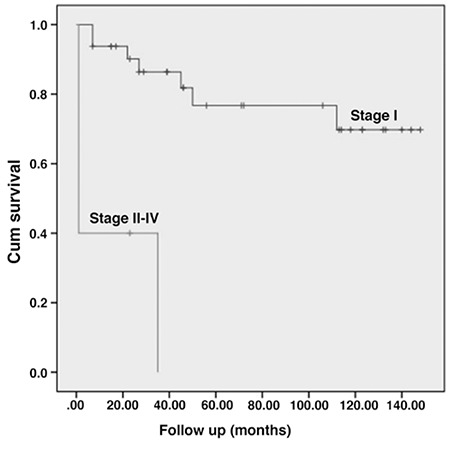
Progression-free survival rates of patients were grouped according to FIGO stage (stage 1 and stage 2-4)

**Figure 2:**
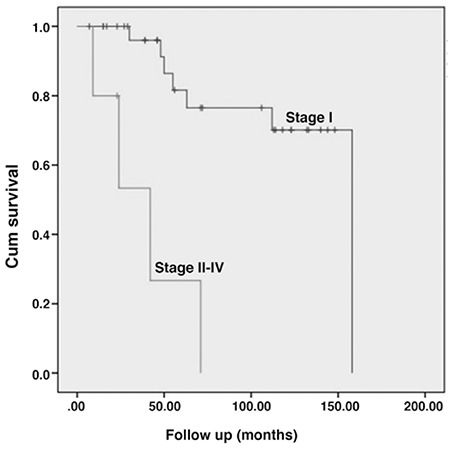
Overall survival rates of patients were grouped according to FIGO stage (stage 1 and stage 2-4)

The median follow-up period was 71 months (range, 1-158 months). Recurrence developed in 5 (13.5%) patients, of whom 4 had stage 1B disease, and 1 had stage 2 disease. There was only one vaginal cuff recurrence. The rest of the recurrences were outside the P cavity (3 had lung recurrence, 1 had both omental and lung recurrences). The median PFS and OS for all patients were 50 months (range, 1-148 months) and 71 months (range, 7-158 months), respectively. The median OSs for women with stage 1 and ≥ stage 2 disease were 59 months (range, 7-158 months) and 24 months (range, 9-71 months), respectively (Figure 1, 2). The 5-year PFS and OS rates for all patients were 68% and 74%, respectively. The 5-year OS rates for women with stage 1 and ≥ stage 2 disease were 82% and 27%, respectively.

## DISCUSSION

ULMSs are rare and rather aggressive tumors that have poor outcomes with early-onset extra-uterine metastases and distant recurrences^(3)^.

Many clinico-pathologic variables such as age at diagnosis, menopausal status, race, stage, grade (nuclear atypia), mitotic counts, PTD, and lymph node status were studied for any potential prognostic impact in women with ULMS. Among these, the most common accepted prognostic factors were stage and nuclear atypia^(5,8,9,10,11,12,13,14,15)^. In the present study, stage 1 disease was found to be an independent favorable prognostic factor for both OS and PFS. However, nuclear atypia did not reach statistical significance as a poor prognostic factor. Mitotic counts >15 per 10 high-power fields (HPF) were reported to be a significant factor of poor prognosis on univariate analysis by Wu et al.^(15)^ Pautier et al.^(11)^ reported that stage and mitotic counts were the only factors that reached statistical significance in predicting both OS and PFS on multivariate analysis. In another study, higher mitotic activities per any HPF were associated with a decrease in survival. Grade did not have an impact on survival and the absence of necrosis was a favorable prognostic feature^(16)^. The association between necrosis and PFS was first described by Hsieh et al.^(8)^ In line with some above mentioned studies, we found that tumor necrosis was associated with poor PFS.

Total abdominal hysterectomy is the standard approach as an initial therapy for ULMS but the need for additional surgical procedures such as BSO, P and/or PA lymphadenectomy and adjuvant therapy is a widely debated issue. Apparently, in line with the results of some studies in the literature, there is no clear benefit in performing salpingo-oophorectomy and/or lymph node dissection in patients with ULMS^(5,6)^. Hsieh et al.^(8)^ observed that relapse occured in 1 out of 5 patients (20%) with stage 1 disease who underwent TAH+BSO and 1 out of 6 patients (16.7%) with stage 1 disease, in whom one or both ovaries were left behind.

The incidence of lymphatic spread is only about 3% in early-stage ULMS^(10,17,18)^. Ayhan et al.^(2)^ reported that neither performing lymphadenectomy nor extent of lymph node dissection (number of resected lymph nodes) has significant effects on both PFS and OS in such patients. However, they emphasized that lymphadenectomy could be considered for patients with extrauterine involvement, clinically suspicious nodes or in postmenopausal women with enlarged uterus or large tumor. In contrast, Leibsohn et al.^(19)^ highlighted the impact of primary tumor diameter and reported a 50% rate of lymph node metastasis in women with tumors measuring 6-10 cm. Kapp et al.^(5^) demonstrated that the incidence of regional lymph node metastasis was low and salpingo-oopherectomy did not improve survival. In the present study, in addition to TAH+BSO, P plus PA lymphadenectomy was performed in 8 (21.6%) patients, and 4 (10.8%) patients underwent only P lymphadenectomy. There were neither P nor PA lymph node metastasis. Additionally, we found no statistically significant difference in terms of survival between patients in the lymphadenectomy and no lymphadenectomy groups.

Abrahao and Maluf reported a case of ULMS that had metastasized to the central nervous system. The authors emphasised that ULMS primarily spreads hematogenously and as in our study, the most common site of distant metastasis was lung. Consequently, a chest X-ray or a computerized tomography should be performed as a part of initial staging^(20)^.

As well as extensive surgery, the role of adjuvant CT is controversial. A study of doxorubicin alone demonstrated no benefit in survival^(21)^. On the other hand, a study by Hensley et al.^(22)^ combination CT with gemcitabine, docetaxel, and doxorubicin has increased expectations of adjuvant CT treatment in such patients. In another study by Wong et al.^(23)^ it was reported that postoperative pelvic RT reduced recurrence and had a significant effect on OS. In contrast, Reed et al.^(24)^ showed that adjuvant RT was not associated with improved survival in women with stage 1 and 2 disease. Additionally, Yu et al.^(25)^ reported that pelvic RT had no beneficial effects on survival and distant control. They also emphasized that CT must be the corner stone of adjuvant therapy. Conversely, in the present study both adjuvant treatment modalities significantly prolonged PFS and OS. Finally, in our study the 5-year PFS and OS rates for all patients were higher (68% and 74%, respectively) than the survival rates in most studies (45-73% and 10-73%, respectively)^(2,3,4,5,9,10,15,16,26,27,28,29)^. This was probably due to the higher proportion of patients who were diagnosed at stage 1.

The limitations of this study are its retrospective nature, and some patients were treated by non-gynecologic oncologic surgeons and therefore patients were treated with different types of surgical approaches over the 15-year time period. Retrospective cohort studies are subject to selection bias, recall bias, and unknown confounding variables, which may negatively impact the accuracy of the results. Moreover, during the 15-year study period, significant improvements in surgical techniques and adjuvant treatment may have also affected the results. Lastly, the data did not allow for definitive and comparative analyses to assess the heterogeneity of the different adjuvant therapy regimens. Despite these limitations, the relatively large number of patients diagnosed as having this rare disease, with similar demographic characteristics was included in this study. In addition, good follow-up data were available. Additionally, the surgeries were performed at a single institution, and all pathologic slides were reviewed by an experienced gynecologic pathologist. All of these factors most likely increased the validity of the results and mitigated the limitations.

In conclusion, our study demonstrated stage 1 disease to be the only independent prognostic factor for survival in women with ULMS. Surgery remains the primary treatment modality. Spread of ULMS is mainly hematogenous so extensive surgery including lymphadenectomy appears to be of less importance. Therefore, quality of life issues, operability, and the most appropriate and effective treatment regimens should also be considered for management. Further improvements in survival rates require the optimization of adjuvant therapy modalities.

## Figures and Tables

**Table 1:**
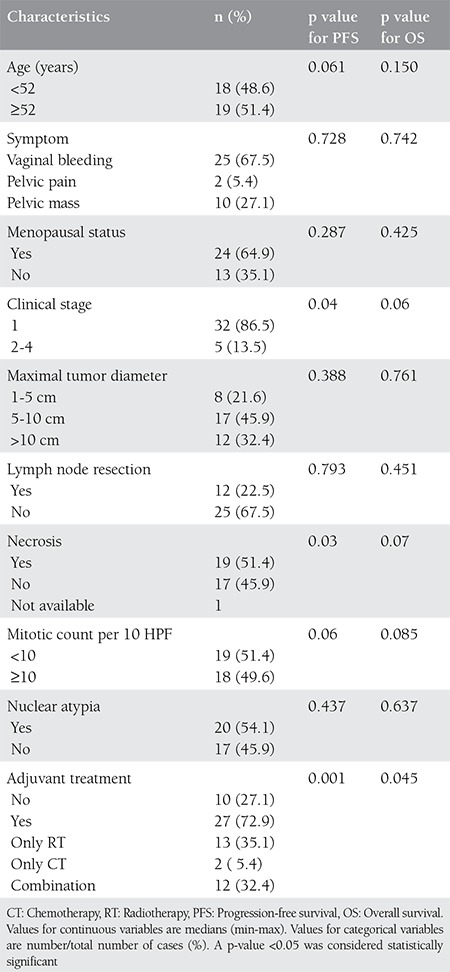
Clinico-pathologic characteristics with respect to progression-free survival and overall survival
